# Molecular Profiling of a Rare Rosette-Forming Glioneuronal Tumor Arising in the Spinal Cord

**DOI:** 10.1371/journal.pone.0137690

**Published:** 2015-09-15

**Authors:** Lucas Tadeu Bidinotto, Cristovam Scapulatempo-Neto, Alan Mackay, Gisele Caravina de Almeida, Bernd Walter Scheithauer, Gustavo Noriz Berardinelli, Raul Torrieri, Carlos Afonso Clara, Leonir Terezinha Feltrin, Marta Viana-Pereira, Marileila Varella-Garcia, Chris Jones, Rui Manuel Reis

**Affiliations:** 1 Molecular Oncology Research Center, Barretos Cancer Hospital, Barretos, SP, Brazil; 2 Barretos School of Health Sciences, Dr. Paulo Prata—FACISB, Barretos, SP, Brazil; 3 Department of Pathology, Barretos Cancer Hospital, Barretos, SP, Brazil; 4 Divisions of Molecular Pathology and Cancer Therapeutics, Institute for Cancer Research, London, Surrey, United Kingdom; 5 Department of Laboratory Medicine and Pathology, Mayo Clinic, Rochester, MN, United States of America; 6 Department of Neurosurgery, Barretos Cancer Hospital, Barretos, SP, Brazil; 7 Department of Radiology, Barretos Cancer Hospital, Barretos, SP, Brazil; 8 Life and Health Sciences Research Institute (ICVS), School of Health Sciences, University of Minho, Braga, Portugal; 9 3B's—PT Government Associate Laboratory, Braga/Guimarães, Portugal; 10 University of Colorado Anschutz Medical Campus, Medical Oncology/Department of Medicine, Aurora, Colorado, United States of America; University Hospital of Navarra, SPAIN

## Abstract

Rosette-forming glioneuronal tumor (RGNT) of the IV ventricle is a rare and recently recognized brain tumor entity. It is histologically composed by two distinct features: a glial component, resembling pilocytic astrocytoma, and a component forming neurocytic rosettes and/or perivascular rosettes. Herein, we describe a 33-year-old man with RGNT arising in the spinal cord. Following an immunohistochemistry validation, we further performed an extensive genomic analysis, using array-CGH (aCGH), whole exome and cancer-related hotspot sequencing, in order to better understand its underlying biology. We observed the loss of 1p and gain of 1q, as well as gain of the whole chromosomes 7, 9 and 16. Local amplifications in 9q34.2 and 19p13.3 (encompassing the gene *SBNO2*) were identified. Moreover, we observed focal gains/losses in several chromosomes. Additionally, on chromosome 7, we identified the presence of the *KIAA1549*:*BRAF* gene fusion, which was further validated by RT-PCR and FISH. Across all mutational analyses, we detected and validated the somatic mutations of the genes *MLL2*, *CNNM3*, *PCDHGC4* and *SCN1A*. Our comprehensive molecular profiling of this RGNT suggests that MAPK pathway and methylome changes, driven by *KIAA1549*:*BRAF* fusion and *MLL2* mutation, respectively, could be associated with the development of this rare tumor entity.

## Introduction

Rosette-forming glioneuronal tumor (RGNT) of the fourth ventricle is a very recent entity, recognized in the latest, 2007 edition, of the WHO classification of Central Nervous System Tumors [1]. RGNT is composed by two distinct features: a predominant glial component, whose morphology resembles pilocytic astrocytoma, and a neurocytic component forming neurocytic rosettes and/or perivascular pseudorosettes. This rare tumor affects predominantly adult females (61%), with mean age around 32 years, typically originating in the fourth ventricle and/or aqueduct [1, 2]. Due to its usual indolent course, it is considered a WHO grade I tumor [1].

Hitherto, less than 100 cases are reported in the literature [3], most of them as case reports of MRI, histopathological and immunohistochemical findings [4–9]. There are few molecular studies, with the only recurrent genetic alterations identified being *PIK3CA* [10, 11] or *FGFR1* mutations [12].

Herein, we describe a 33-year-old man with RGNT arising in a peculiar location, namely the spinal cord. We further performed immunohistochemistry and molecular analysis, using array-CGH, whole exome and cancer-related hotspot sequencing, in order to better understand its underlying biology.

## Material and Methods

A 33-year-old male was operated on Barretos Cancer Hospital, to resect a tumor located in the spinal cord. Following gross-total resection, part of the lesion was formalin-fixed and paraffin embedded for standard H&E and immunohistochemistry staining, and the remaining tissue was snap frozen for further molecular analysis. Additionally, peripheral blood was collected. The individual in this manuscript has given written informed consent (as outlined in PLOS consent form) to publish these case details. The local Ethical Committee of Barretos Cancer Hospital has approved this study under the processes number 262/2009 and 408/2010.

Immunohistochemical analyses (streptoavidin-biotin peroxidase method) were performed for specific markers, according to the S1 Table. The reactions were performed using the Ventana System (Ventana Systems Inc.), following suppliers’ recommendations.

Microsatellite instability analysis in tumor and blood DNA was performed according to Viana-Pereira et al [13]. For aCGH, two-color Comparative Genomic Hybridization microarray was performed using default Agilent enzymatic labeling protocol. Four hundred nanograms of both tumor and blood (used as reference) DNA were digested by *AluI* and *RsaI* restriction enzymes, and incubated with random primers. Blood DNA was labeled with cyanine-3, whereas tumor DNA was labeled with cyanine-5. Labeled DNAs were hybridized into Agilent Human Genome CGH 8x60K microarray slide, and washed according to supplier’s protocol. The slide was scanned and decoded by the software Feature Extraction v.10.7 (Agilent Technologies). The signal intensities were log2 transformed, and the spots mapped to hg19. Data were Lowess normalized, smoothing corrected, and CBS segmented. aCGH data can be accessed using the Gene Expression Omnibus number GSE64891.

For known cancer-related hotspot mutational screening, Ion Torrent platform (Life Technologies) was used. The library was built according to the default protocol described by the supplier. Ten nanograms of tumor DNA was amplified using Ion AmpliSeq Library kit 2.0 (Life Technologies) and Ion AmpliSeq Cancer Primer Pool. After sample tracking preparation using Ion AmpliSeq Sample ID Panel, the positive Ion Spheres were enriched by Ion PGM Template One Touch 200 kit and further sequenced in an Ion 316 chip, using Ion PGM Sequencing 200 kit v2. The analysis was performed based on coverage and reads quality, and frequency of reference/variant bases in each position, using the software Ion Torrent Variant Caller v.3.6.2.

Exome sequencing was performed on tumor and peripheral blood DNA. Briefly, exome capture was carried out using the 50Mb Agilent SureSelect platform (Agilent Technologies), and paired-end-sequenced on an Illumina HiSeq2000 (Illumina Inc.) with a 100bp read length. Reads were mapped to the hg19 build of the human genome using bwa, and PCR duplicates removed with PicardTools 1.5. Somatic single nucleotide variants were called using the Genome Analysis Tool Kit v.2.4–9. Variants were annotated using the Ensembl Variant Effect Predictor v.71 incorporating SIFT and PolyPhenpredictions, COSMIC v64 and dbSNP build 137 annotations.

In order to validate the mutations identified through Exome NGS, primers for all somatic variants were designed and PCR [14] followed by direct sequencing was performed (S2 Table). Additionally, the most relevant hotspots regions of *PIK3CA* were assessed using the oligonucleotide primers (S2 Table). The PCR reactions of *PIK3CA* were performed in a final volume of 15 μL, under the conditions: 7.2 μL HotStar Master Mix (QIAGEN), 5.6 μL H2O2 (QIAGEN), 0.3 μL each primer and 0.6 μL MgCl2 5 mM. PCR amplification was performed in a Veriti 96 Well Thermal Cycler (Applied Biosystems) with an initial denaturation step at 96°C for 15’, then amplified for 40 cycles of denaturation at 96°C for 45s, annealing at 55.5°C for 45s, extension at 72°C for 45s and final extension at 72°C for 10’.

After amplification, the PCR products were firstly purified with EXO-SAP (GE Techonology), and then submitted to a sequencing reaction using 1 μL of BigDye Terminator v3.1 (Applied Biosystems), 1.5 μL of sequencing buffer and 1 μL of each primer. The sequencing reaction was followed by post sequencing purification with BigDyeXTerminator Purification Kit following the manufacturer’s instructions. Direct sequencing was performed in 3500xL Genetic Analyzer (Applied Biosystems).

For mRNA analysis, first-strand cDNA was synthetized from five hundred nanograms of tumor RNA using SuperScript III First-Strand Synthesis SuperMix (Invitrogen), according to the protocol provided by the supplier, using random hexamers. The nested PCR was performed using 2 pairs of primers specially designed for detecting the fusion *KIAA1549*:*BRAF*, according to Forshew et al. (2009) [15] (S2 Table). Finally, the PCR product was purified and sequenced.

*KIAA1549*:*BRAF* gene fusion was also evaluated by FISH, as previously described [16]. Briefly, FFPE sections were incubated at 56°C for 2 h, dewaxed in CitriSolv (Fisher), air dried, and dehydrated. Pre-hybridization procedure was performed with reagents from the SPOT-Light Tissue Pretreatment Kit (Invitrogen). Specimen were boiled in heat pretreatment solution for 50’, washed in PBS, digested with the enzyme reagent for 55’ at 37°C, washed in PBS, dehydrated and air dried. The KIAA1549/BRAF probe mix (200ng of BRAF SR DNA–BAC clone RP4-726N20—and 200ng of KIAA1549 SG DNA–BAC clone RP11-148L5) was applied to the selected hybridization area, which was covered with a coverslip and sealed with rubber cement. DNA co-denaturation was performed in the HYBrite (Vysis) at 85°C for 5’ and hybridization was allowed to occur at 38°C for 40 h. Post-hybridization washes were performed by incubating in 2×SSC/0.3% NP-40 at 74°C for 3’ then in 2×SSC at room temperature for 2’, followed by dehydration. Finally, DAPI/anti-fade (0.3ug/ml in Vectashield mounting medium) was applied and the area covered with a coverslip.

Analysis was performed on epifluorescence microscope using single interference filter sets for green (FITC), red (Texas red) and blue (DAPI) as well as dual (red/green) and triple (blue, red and green) band pass filters. Fifty nuclei per specimen were analyzed for tandem duplication of KIAA1549 and BRAF, which was identified as presence of overlapping red and green fluorescent signals.

## Results

**Fig 1:**
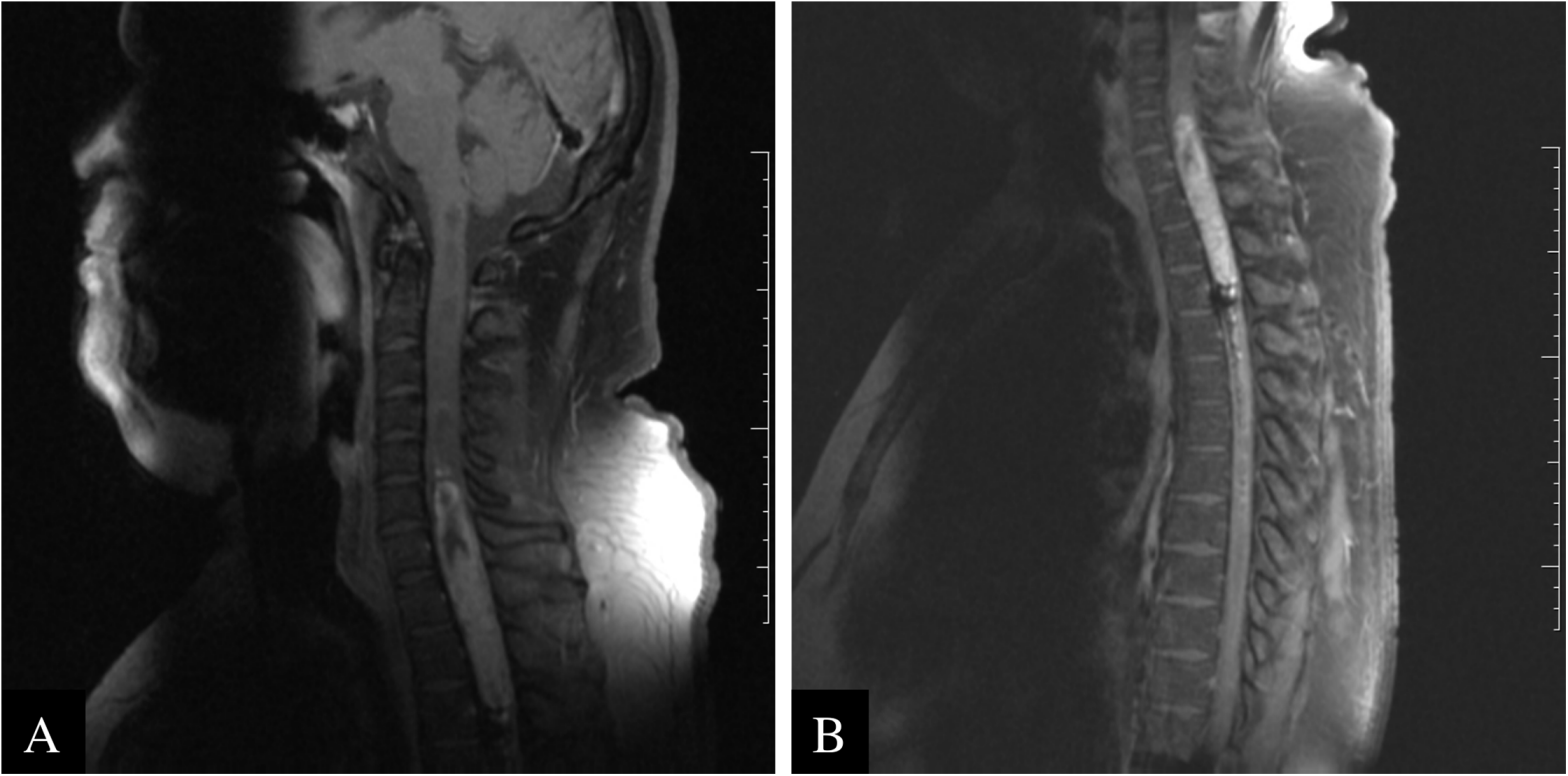
Imaging features of the RGNT. (A and B) Magnetic resonance images showing an expansive lesion between C6 and T3.

The patient presented with progressive gait disturbance, thoracic spine pain and bladder dysfunction. Physical examination revealed weakness of the upper and lower extremities and spasticity with clonus and exaggerated muscle stretching reflexes. A spinal MRI scan with gadolinium revealed a contrast enhancing, expansive lesion centrally located in the cervical-thoracic spinal cord (C6 to T3), and measuring 9.5x1.9x1.5cm, with extended seringomielin and hemosiderin (Fig 1).

A C6-T4 laminectomy and gross-total resection was undertaken. The patient was discharged after 18 days, and clinical examinations and MRI were performed every 6 months. In the last follow up (January 23^rd^ 2014, 52 months after the surgery), besides the latent paraplegia, with sensitive level up to T4, and urinary bladder dysfunction, the patient was alive with no abnormalities.

### Histopathological and immunohistochemical characterization

**Fig 2:**
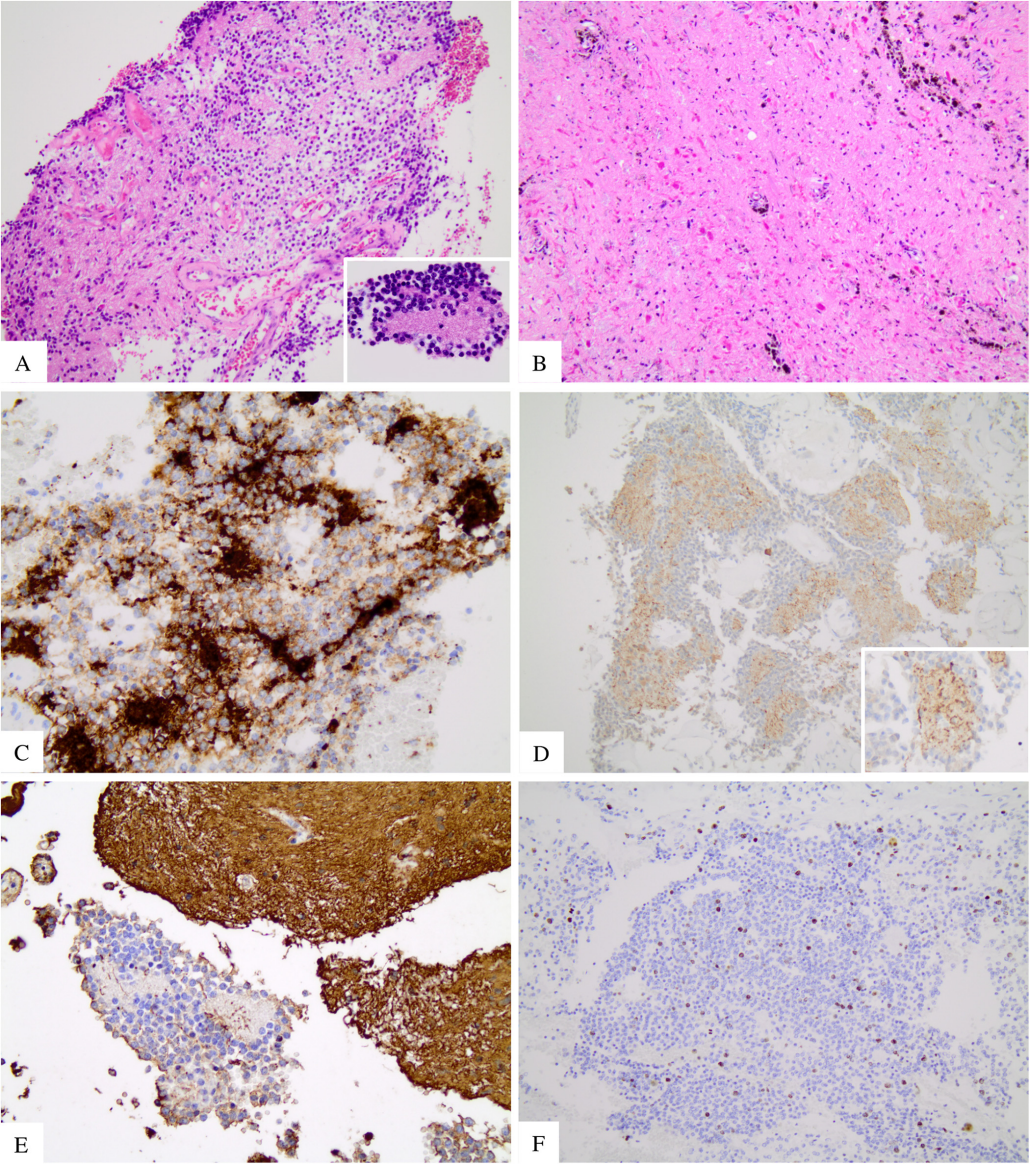
Pathologic and immunohistochemistry features of the RGNT. (A) HE showing the neurocytic and glial area (H&E 200x). Inset detailing the neurocytic rosette with a neuropil core (H&E 1000x). (B) Glial component (H&E 200x). (C) Neurocytic component positive for synaptophysin (400x). (D) Neurocytic component stained by MAP2 expression (200x) that highlights the neuropil of the neurocytic rosette in detail (400x). (E) Neurocytic area negative for GFAP and glial area positive for GFAP (400x). (F) Ki67 showing low proliferation index in neurocytic area (200x).

The histology revealed a WHO grade I RGNT typically found in the fourth-ventricle. This tumor featured the usual biphasic neurocytic (Fig 2A) and glial (Fig 2A and 2B) architecture. The neurocytic component consisted of a uniform population of neurocytes forming neurocytic rosettes, characterized by a ring of neurocytic cells with small and monomorphic nuclei with an eosinophilic and acellular neuropil core. Vascular pseudorosettes were also present (Fig 2A inset). The glial component was dominant, and resembled pilocytic astrocytoma composed of spindle to stellate in shape with elongate to oval nuclei and moderately dense chromatin. Some cells superficially resembled oligodendroglia cells with occasional perinuclear halos. Rosenthal fibers, hemosiderin deposits associated with thick-walled, and occasionally hyalinized vessels were observed. Immunoreactivity for synaptophysin (Fig 2C), neurofilament and MAP2 (Fig 2D) proteins were depicted in the neurocytic component, whereas the glial component exhibited positivity for GFAP (Fig 2E) and S-100. Neurocytic component was found negative for GFAP (Fig 2E). Mitoses were not observed and the proliferation index labeled by Ki-67 expression was observed in approximately 3% of cells (Fig 2F). There was no necrosis, anaplasia or microvascular proliferation. Due to the rarity of this lesion, and its atypical location, the case was further sent to an expert in neuropathology (B.W.S.), for a second opinion, who confirmed the initial diagnosis of RGNT.

### Molecular profile

In order to better characterize the genetic alterations of this rare case, we performed an extensive analysis of genetic instability, either at the chromosomal (aCGH) and the nucleotide level (microsatellite instability), screened for the presence of mutations in 46 major cancer related genes, and extended the analysis to whole exome sequencing.

**Fig 3:**
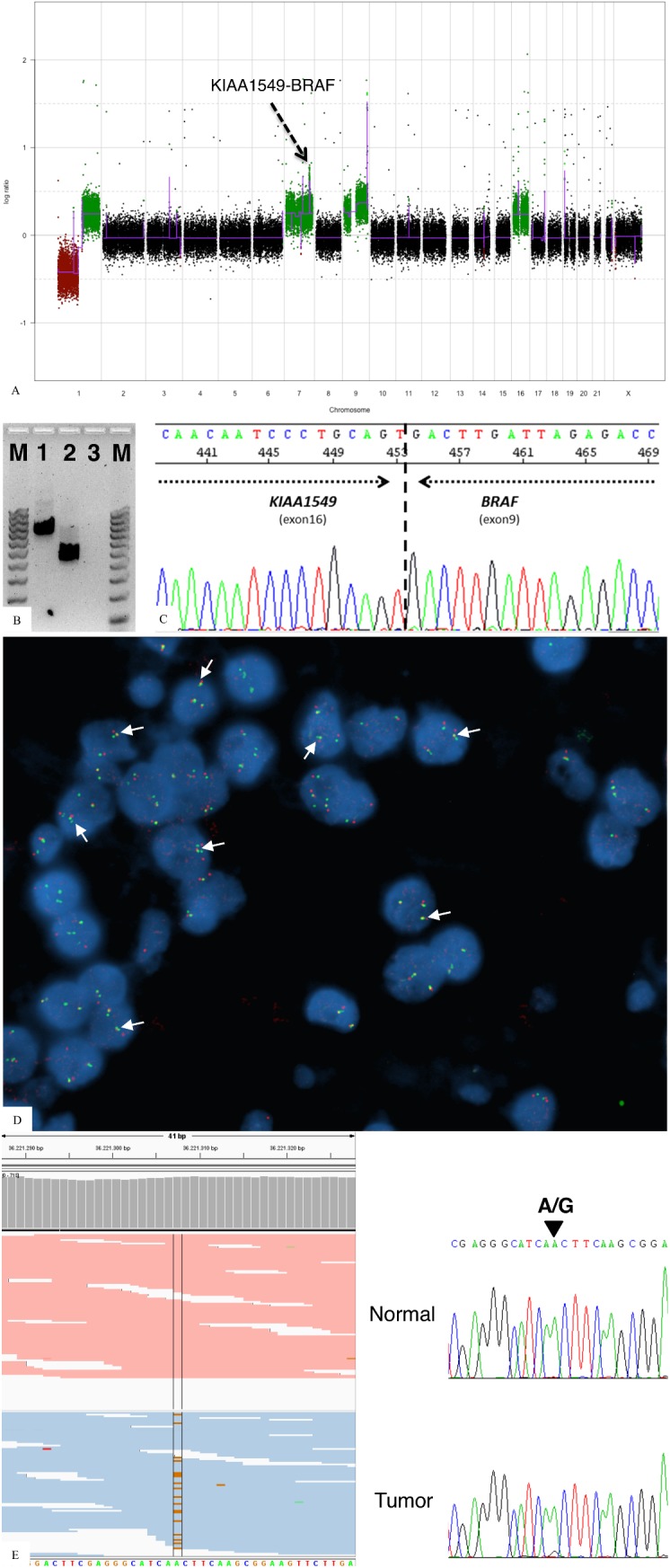
Molecular features. (A) Plot representing the whole genome, highlighting the KIAA1549:BRAF fusion (dashed arrow), (B) Agarose gel representing: M. 100 bp ladder; 1. RT-PCR result using the pair of primers for detecting *KIAA1549*:*BRAF* fusion; 2. the housekeeping gene *GAPDH*; 3. negative control. C) Direct sequencing of the band of the Fig 3A.1, showing the fusion point of *KIAA1549* (exon 16) and *BRAF* (exon 9). D) FISH results of the glioneuronal portion of the tumor showing yellow signals, representing the overlapping of *KIAA1549* (green) and *BRAF* (red) signals (arrows). E) IGV visualization of exome NGS results (left) and direct sequencing validation (right) of *MLL2* gene.

Array-CGH showed loss of 1p and gain of 1q, as well as gain of the whole chromosomes 7, 9 and 16 (Fig 3A). Moreover, we observed focal gains/losses in the chromosomes 1, 2, 3, 6, 7, 11, 14, 17, 22 and X, and local amplifications in 9q34.2 and 19p13.3 (S3 Table). A higher level of gain was observed at chromosome 7q34, encompassing the genes *KIAA1549* through *BRAF*, with breakpoints in those genes (Fig 3A–dashed arrow). RT-PCR was performed to confirm that this genomic gain reflected the fusion of the genes *KIAA1549*:*BRAF*. The RT-PCR showed a band of the expected size (approximately 800bp) (Fig 3B). To further verify the presence of *KIAA1549*:*BRAF* gene fusion, the RT-PCR product was sequenced and the fusion between exon 16 of *KIAA1549* and exon 9 of *BRAF* gene was detected (Fig 3C). Finally, we performed FISH to confirm the presence of the fusion (Fig 3D). As observed, the overlapping signals of *KIAA1549* (green) and *BRAF* (red) generated by the tandem duplication, observed as red-green doublets or yellow spots, denotes the presence of *KIAA1549*:*BRAF* gene fusion (Fig 3D). Of note, the positive pattern for *KIAA1549*:*BRAF* fusion was found only in the neurocytic component, whereas the cells in the areas consistent with the glial component were negative.

The tumor exhibited a microsatellite stable (MSS) phenotype (data not shown). Previously reported recurrent *PI3KCA* and *FGFR1* hotspot mutations were not detected by direct sequencing and Ampliseq Cancer. Somatic mutations were identified in the genes *MLL2* (Fig 3E), *CNNM3*, *PCDHGC4* and *SCN1A* (Table 1) by whole exome sequencing and validated by direct sequencing.

**Table 1:** Somatic mutations validated by direct sequencing.

Chromosome	Position	Gene	Ref^a^	Var^a^	Aminoacid	Zygosity	Functional effect prediction
2	97483123	*CNNM3*	T	G	F370C	Heterozygous	Possibly damaging
2	166897773	*SCN1A*	GGTCCGTCATTGGATAGTGC	G	^b^	Heterozygous	NA^c^
5	140866746	*PCDHGC4*	C	T	T669I	Heterozygous	Benign
19	36221307	*MLL2*	A	G	N1714S	Heterozygous	Possibly damaging

^a^Ref represents the reference allele and Var represents the variant allele.

^b^Frameshift mutation.

^c^NA = Information not available

## Discussion

This study describes for the first time a comprehensive genetic characterization of a case of RGNT of the fourth ventricle arising in the spinal cord. This is not the usual presentation, yet it has been previously reported [17, 18]. The MRI images showed a relatively well-demarcated lesion, with low and high intensities in T1 and T2, respectively, which corroborate findings of other studies [8, 17, 18].

The presentation of this case could resemble other tumor types described in the literature, such as glioneuronal tumor with neuropil islands (GNTNI), a WHO grade II/III that affects the spinal cord in in 23% of cases [3]. GNTNI is an infiltrative astrocytoma with scarce micronodules (islands of neuropil) or bigger and atypical islands delimitated by oligodendrocyte-like cells that stains for synaptophisin or Neu-N. The glial component of GNTNI is composed predominantly by a astroglial fibrillary or gemistocytic component, and not by a pilocytic component as find in RGNT, and this glial component can be atypical with increased mitotic activity, microvascular proliferation and necrosis. In the present case, the morphological diagnosis of RGNT at H&E-stained slides was further confirmed by immunohistochemistry. The neurocytic component was positive for synaptophisin, neuron specific enolase and MAP2, and the glial component was positive for GFAP and S-100 protein. Furthermore, no mitotic activity was observed. These morphological and immunohistochemistry differences were recognized and utilized to rule out the diagnosis of GNTNI, and confirm the diagnosis of RGNT.

In order to interrogate the genetic abnormalities of this rare case, we analyzed the presence of chromosomal and microsatellite instability. We found loss in 1p, gain in 1q, 7, 9 and 16. There are two studies that evaluated the co-deletion 1p/19q, and no alterations were found [19, 20]. Interestingly, we observed loss of the entire short arm of chromosome 1. Of particular interest was the observed gain of chromosomes 9 and 16, events not frequently described in SNC tumors [21]. Additionally, we found focal amplification at 19p13.3, where the *SBNO2* gene is located. This gene codifies a protein that exhibits a RNA helicase activity [22], and is involved in anti-inflammatory responses, regulated by IL-10 in a STAT3-dependent way [23]. To date, there are no reports relating directly this specific gene to cancer development. Further studies are needed to understand whether *SBNO2* gene amplification leads to protein overexpression, and gain of activity in RGNTs.

Importantly, we observed a gain in the chromosome 7 encompassing the genes *KIAA1549* and *BRAF*, with intragenic breakpoints in both genes. We performed RT-PCR to validate the presence of a fusion, which was further confirmed by Sanger sequencing and FISH, which highlighted a fusion between the exon 16 of *KIAA1549* and the exon 9 of *BRAF*. The presence of *KIAA1549*:*BRAF* fusion is found in 60% of pilocytic astrocytomas [16] and was not previously detected in RGNT in the literature [24–26]. Morphologically, the glial component of both tumors are identical, and could explain the finding of *KIAA1549*:*BRAF* fusion, that is frequently found in pilocytic astrocytomas. Interestingly, we observed the presence of the *KIAA1549*:*BRAF* gene fusion only in the neurocytic component.

We further performed extensive mutation profiling, corroborating recent studies that reported the absence of mutations in the *BRAF*, *IDH1* and *IDH2* genes [24, 27], and further failed to identify the presence of *PI3KCA* gene mutations as suggested by Ellezan and collaborators [10], nor *FGFR1* gene mutations as suggested by Gessi and collaborators [12]. Importantly, whole exome sequencing identified somatic mutation in four genes—*MLL2*, *CNNM3*, *PCDHGC4* and *SCN1A*. Among these genes, we found *MLL2* to be of particular interest. *MLL2*, mapped on chr19q13.12, is described to be required for the maintenance of basal transcription machinery stabilization [28]. Loss-of-function mutations of this gene have been present in the genetic landscape of meduloblastoma [29, 30], since it is able to change the gene expression pattern by impairing H3K4me1/2 on genes enhancers [31].

In conclusion, our comprehensive molecular profiling of a RGNT case suggests the existence of a unique genetic pathway for the development of these tumors: *KIAA1549*:*BRAF* fusion is a possible driver by constitutively activating MAPK pathway, [32] and *MLL2* mutation may lead to profound changes in the transcriptome [33]. Whole genome sequencing studies of low-grade gliomas suggest that few genetic alterations are required for oncogenesis, and there are some recurrent chromosomal abnormalities, depending on the histopathological subtype [33]. Taken together, these mechanisms may increase survival and/or tumorigenic capacity of cells, leading to the development of this rare entity.

## Supporting Information

S1 Table Primary antibodies and experimental details used for immunohistochemistry analysis. (DOCX)

S2 Table Primers used in direct sequencing validation and KIAA1549:BRAF fusion. (DOCX)

S3 Table Regions presenting point copy number gains and losses. (DOCX)
