# Supplementary material for: Molecular Profiling of a Rare Rosette-Forming Glioneuronal Tumor Arising in the Spinal Cord
Source: PLoS One. 2015 Sep 15;10(9):e0137690. doi: 10.1371/journal.pone.0137690 (PMC4570813; doi:10.1371/journal.pone.0137690)
Supplement: S1 Table — (DOCX) [file pone.0137690.s001.docx]

Supplementary Table 1 – Primary antibodies and experimental details used for immunohistochemistry analysis.

| Primary antibody | Dilution | Company |
| --- | --- | --- |
| Ki-67 | 1:400 | Cell Marque Corporation (Rocklin, CA, USA) |
| Synaptophysin | 1:300 | Spring Bioscience (Pleasanton, CA, USA) |
| Neurofilament | 1:200 | Cell Marque Corporation (Rocklin, CA, USA) |
| GFAP | 1:100 | Dako (Carpinteria, CA, USA) |
| S-100 | 1:500 | Diagnostic Biosystems (Pleasanton, CA, USA) |
