# Supplementary material for: Molecular Profiling of a Rare Rosette-Forming Glioneuronal Tumor Arising in the Spinal Cord
Source: PLoS One. 2015 Sep 15;10(9):e0137690. doi: 10.1371/journal.pone.0137690 (PMC4570813; doi:10.1371/journal.pone.0137690)
Supplement: S2 Table — (DOCX) [file pone.0137690.s002.docx]

Supplementary Table 2 – Primers used in direct sequencing validation and KIAA1549-BRAF fusion

| Region | Gene name | Primer sequence |
| --- | --- | --- |
| chr1:16070500-16070737 | *TMEM82* | F - 5'-GTGAGAGAGATGATCCCCTAGG-3' |
|  |  | R - 5'-CTCAGGCCACAGGTCAGC-3' |
| chr2:97483084-97483247 | *CNNM3* | F - 5'-CCAACATCGTGGACATGCTC-3' |
|  |  | R - 5'-GCCGTTACCTCGCTTGAATT-3' |
| chr2:141294125-141294323 | *LRP1B* | F - 5'-GAGCAAACCCATATTGGACACT-3' |
|  |  | R - 5'-TTTGCCATTGCACAAAAGTATT-3' |
| chr2:166897642-166897854 | *SCN1A* | F - 5'-TGAAGGATGGTTGAAAGACTGC-3' |
|  |  | R - 5'-CCATTTGTTGACCTGGCCAT-3' |
| chr2:233198620-233198787 | *DIS3L2* | F - 5'-CCCTGTACACACACTTCACC-3' |
|  |  | R - 5'-AGAATGGTCTTGGGCCACT-3' |
| chr3:121838313-121838543 | *CD86* | F - 5'-GATGAAGCCCAGCGTGTTTT-3' |
|  |  | R - 5'-TGGAGTTACAGGGAGGCTAT-3' |
| chr5:140866647-140866876 | *PCDHGC4* | F - 5'-CTGACCTCCCACCACAGAAG-3' |
|  |  | R - 5'-GAGTAGTGCCACGAATGAGC-3' |
| chr5:169535539-169535724 | *FOXI1* | F - 5'-GCCAATTCAGCCCTCACTTC-3' |
|  |  | R - 5'-GCTTATGTCTGGGCAGTTCC-3' |
| chr7:102279536-102279735 | *UPK3BL* | F - 5'-CCCCTACTGTTTGACCGGAG-3' |
|  |  | R - 5'-GACTCCAGGGGACAGACTTG-3' |
| chr8:72951813-72952040 | *TRPA1* | F - 5'-GCATTTGCCTTACTTATTTCTTCA-3' |
|  |  | R - 5'-TTTCTCAGGCAATGGTACAAAA-3' |
| chr10:70742144-70742387 | *DDX21* | F - 5'-TGATGGGTGGCCAGTGTTAT-3' |
|  |  | R - 5'-AAGCCTCCATATCCTTCCCG-3' |
| chr10:75236868-75237036 | *PPP3CB* | F - 5'-AAGCCTCAGGTTCCTCATCT-3' |
|  |  | R - 5'-GAGGTAGAATTGTGGGGTCAAAT-3' |
| chr11:67173243-67173431 | *TBC1D10C* | F - 5'-CCCAGCTCTACAGTCTTGCA-3' |
|  |  | R - 5'-GCCCTGCTCCGGTCGATA-3' |
| chr14:19685191-19685340 | *AL589743.1* | F - 5'-GGGGAGAAGCTGGCACTG-3' |
|  |  | R - 5'-ACCTGCTTCTTCTCTTGGCT-3' |
| chr14:24458103-24458346 | *DHRS4L2* | F - 5'-CTACTCTGTCACCTCCGCTG-3' |
|  |  | R - 5'-CGAGACACCAGTGCATGTTT-3' |
| chr15:29418465-29418714 | *FAM189A1* | F - 5'-CCTACCTGCCATGCTAGATGT-3' |
|  |  | R - 5'-CCATGTCCCTTCTCTTGCAG-3' |
| chr15:51868361-51868579 | *DMXL2* | F - 5'-ACAGCCTGATCCATATGCCT-3' |
|  |  | R - 5'-GCATGGAGTGTCATCAGTTTATT-3' |
| chr17:26116510-26116714 | *NOS2* | F - 5'-CCACTCTCCATCCAAACCCT-3' |
|  |  | R - 5'-TCCAGTGACACAGGATGACC-3' |
| chr17:48460276-48460513 | *LRRC59* | F - 5'-TGTGTCCAGCAGAACCCAAT-3' |
|  |  | R - 5'-CTGCTGCTATTTGGTGTGGC-3' |
| chr17:78971094-78971275 | *CHMP6* | F - 5'-CAGGAGGATGAAGACGCCAT-3' |
|  |  | R - 5'-CCAGTCACAAACCGCAGAC-3' |
| chr18:76753507-76753752 | *SALL3* | F - 5'-GAGAAGCCCGTGACCACC-3' |
|  |  | R - 5'-GAGCGACTGTGGGGACTC-3' |
| chr19:651552-651791 | *RNF126* | F - 5'-GCCAGAACTTCCGACCTCAA-3' |
|  |  | R - 5'-ATGACAGCTTCGAGATCCCC-3' |
| chr19:7809721-7809921 | *CD209* | F - 5'-CAAGTGGAGCAAAACCCCTC-3' |
|  |  | R - 5'-TCATGTCTAACTCCCAGCGG-3' |
| chr19:11257902-11258127 | *SPC24* | F - 5'-AATTTCCTGGAGAGCTGGGT-3' |
|  |  | R - 5'-GAGGTGGAGGTTGCAATGAG-3' |
| chr19:36221239-36221457 | *MLL2* | F - 5'-CCAGGAAATTGTGAACCCCG-3' |
|  |  | R - 5'-GAGTCAATGCGGATGGAACC-3' |
| chr19:48281982-48282190 | *SEPW1* | F - 5'-CAGGTGGGAGGTTAGTGTGG-3' |
|  |  | R - 5'-GCTCCCTTTTCCCATACAAGG-3' |
| chr20:3675463-3675625 | *SIGLEC1* | F - 5'-ACCAACGATATGAGGTCCCC-3' |
|  |  | R - 5'-AGACTGTGAGAGGTGTGGTG-3' |
| *PI3KCA* exon 9 | *PI3KCA* | F - 5'-CTGTGAATCCAGAGGGGAAA-3’ |
|  |  | R - 5’–ACATGCTGAGATCAGCCAAAT-3' |
| *PI3KCA* exon 20 | *PI3KCA* | F - 5’-ATGATGCTTGGCTCTGGAAT-3’ |
|  |  | R - 5’-GGTCTTTGCCTGCTGAGAGT-3' |
| KIAA1549 - NF1^a^ | *KIAA1549* | F - 5'-GAGGGACGCAGGAGATAAGA-3' |
| BRAF - NR1 ^a^ | *BRAF* | R - 5'-AAGTAATCCATGCCCTGTGC-3' |
| KIAA1549 - NF2 ^a^ | *KIAA1549* | F - 5'-CCAGGAAGAGCTCACGGATA-3' |
| BRAF - NR2 ^a^ | *BRAF* | R - 5'-TGCTGAGGTGTAGGTGCTGT-3' |

^a^ Nested PCR
