# Supplementary material for: Molecular Profiling of a Rare Rosette-Forming Glioneuronal Tumor Arising in the Spinal Cord
Source: PLoS One. 2015 Sep 15;10(9):e0137690. doi: 10.1371/journal.pone.0137690 (PMC4570813; doi:10.1371/journal.pone.0137690)
Supplement: S3 Table — (DOCX) [file pone.0137690.s003.docx]

Supplementary Table 3 – Regions presenting point copy number gains and losses.

| Event | Region | Start | End | Genes^b^ |
| --- | --- | --- | --- | --- |
| Amp^a^ | 9q34.2 | 136461184 | 136472255 | NA |
| Amp^a^ | 19p13.3 | 1162640 | 1162699 | *SBNO2* |
| Gain | 1p22.1 | 93417665 | 93417724 | *FAM69A* |
| Gain | 2p24.1 | 20265442 | 20288373 | NA |
| Gain | 3q26.31 | 174021038 | 174021097 | NA |
| Gain | 6q27 | 169943791 | 170469193 | *WDR27, C6orf120, PHF10, TCTE3, C6orf70* |
| Gain | 11q13.3 | 69191919 | 69191978 | NA |
| Gain | 14q24.2 | 72495492 | 72495551 | *RGS6* |
| Gain | 17p13.1 | 7247242 | 7328253 | *ACAP1, KCTD11, TMEM95, TNK1, PLSCR3, C17orf61, NLGN2, SPEM1* |
| Gain | 17q25.3 | 76714629 | 76893023 | *CYTH1, USP36, TIMP2* |
| Gain | 17q25.3 | 77546256 | 77564174 | NA |
| Gain | 22q13.32 | 48697338 | 48880215 | NA |
| Gain | 23q28 | 152314077 | 152855981 | *PNMA6A, PNMA6B, MAGEA1, ZNF275, TREX2, HAUS7, BGN, ATP2B3, FAM58A* |
| Loss | 3q29 | 196583022 | 196653691 | *SENP5* |
| Loss | 7q21.13 | 89703670 | 89703729 | NA |
| Loss | 14q24.2 | 72534704 | 72964922 | *RGS6* |
| Loss | 22q13.32 | 48901041 | 48909049 | *FAM19A5* |
| Loss | 23p22.33 | 939030 | 1795753 | *CRLF2, CSF2RA, IL3RA, SLC25A6, ASMTL, P2RY8, SFRS17A, ASMT* |
| Loss | 23q23 | 115502792 | 115502851 | NA |

^a^Amplifications; ^b^NA represents regions with no genes mapped
